# Saliency-Based Gaze Visualization for Eye Movement Analysis †

**DOI:** 10.3390/s21155178

**Published:** 2021-07-30

**Authors:** Sangbong Yoo, Seongmin Jeong, Seokyeon Kim, Yun Jang

**Affiliations:** Computer Engineering and Convergence Engineering for Intelligent Drone, Sejong University, Seoul 05006, Korea; usangbong@gmail.com (S.Y.); qnqbw@naver.com (S.J.); ksy0586@gmail.com (S.K.)

**Keywords:** gaze data visualization, saliency analysis, visual attention

## Abstract

Gaze movement and visual stimuli have been utilized to analyze human visual attention intuitively. Gaze behavior studies mainly show statistical analyses of eye movements and human visual attention. During these analyses, eye movement data and the saliency map are presented to the analysts as separate views or merged views. However, the analysts become frustrated when they need to memorize all of the separate views or when the eye movements obscure the saliency map in the merged views. Therefore, it is not easy to analyze how visual stimuli affect gaze movements since existing techniques focus excessively on the eye movement data. In this paper, we propose a novel visualization technique for analyzing gaze behavior using saliency features as visual clues to express the visual attention of an observer. The visual clues that represent visual attention are analyzed to reveal which saliency features are prominent for the visual stimulus analysis. We visualize the gaze data with the saliency features to interpret the visual attention. We analyze the gaze behavior with the proposed visualization to evaluate that our approach to embedding saliency features within the visualization supports us to understand the visual attention of an observer.

## 1. Introduction

The human eye selects information through a visual attention competition even if it sees much information at the same time [1]. Gaze behavior is one of the most accessible tools to identify visual attention. Scientists studying the visual attention analyze visual stimuli and generate saliency maps from the visual stimuli [2]. Then, the salient area is measured using the generated saliency map to predict regions with high visual attention.

Gaze behavior is greatly influenced by cognitive controls such as task, memory, and knowledge, but visual saliency significantly influences finding salient areas within the information [3]. The visual saliency is a stimulus-driven signal indicating that the reference position is sufficiently different from the surrounding environment [2,4]. The feature maps are required to create a saliency map for the visual salience computation based on the feature integration theory [5]. Saliency maps [2] are generated with various features, such as intensity, color, orientation [6], log spectrum [7], entropy [8], curvature [9,10], histogram of oriented gradients [11,12,13], and center-bias [14]. The saliency features have also been utilized to predict gaze distributions using the core principle that human visual attention is significantly affected by the salient area [6,15,16,17,18]. However, the predicted gaze using only visual stimuli does not reveal which saliency feature has contributed to the gaze distribution.

In general, eye movements are analyzed through statistical techniques [19,20,21]. Gaze data analysis aims to interpret gaze behavior and human visual attention, and gaze data visualization is a tool for intuitively analyzing gaze behavior. Gaze data visualization is utilized in conjunction with statistical techniques to gain additional insight [22,23]. Most gaze data visualization studies discover rationales related to gaze behavior from cognitive control and analyze common eye movements or personal biases of observers [24,25], essential information from AOIs (areas of interest) [26], the influence of memory and knowledge on eye movements [27], and social communication [28]. According to the analysis of visual attention and eye movement, gaze behavior is affected not only by cognitive control but also by visual saliency [29,30]. In order to use visual saliency for gaze behavior analysis, researchers need to statistically calculate saliency feature values in the fixations separately from gaze data visualization [31] or to utilize tools that show saliency features of visual stimuli [15]. In the existing gaze behavior analysis studies with the visual saliency, statistical analysis of gaze data and saliency features are presented in tables or graphs [6]. Additionally, eye movement data and saliency map are visualized separately [32] or jointly by overlapping eye movement data on the saliency map. However, the existing methods have the inconvenience of seeing the statistical analysis result or the saliency map alternately. Additionally, the overlapping technique is not intuitive because the eye movement data obscure the saliency map used in the analysis. Therefore, to achieve additional insight in gaze behavior analysis, it is necessary to devise a new visualization that intuitively reveals eye movements and visual saliency unitedly.

In this paper, we propose a novel gaze visualization technique that enables to analyze the gaze movements according to the visual attention by employing the saliency features of visual stimuli. Our proposed technique is a saliency-based gaze visualization embedding the saliency features for intuitively revealing knowledge within the gaze data.

We visualize the most prominent saliency features and their ratios with the gaze movement data of an observer. We analyze the gaze data with the visual attention in the case studies. The contributions of our work are as follows:

- We propose a novel gaze data visualization technique embedding the saliency features of visual stimulus as visual clues for analyzing the visual attention of an observer.
- We evaluate whether our gaze visualization benefits to understanding the visual attention of an observer.

Our research goal is to analyze gaze behavior using visual stimulus saliency features as visual clues. Therefore, the main contribution of our paper is the gaze visualization that provides gaze behavior analysis using saliency features. Note that this paper has been extended based on the poster paper published in PacificVis [33]. We added more material for data processing in Section 3, gaze data visualization in Section 4, case studies and user study in Section 5, and the discussion in Section 6.

## 2. Related Work

In the early days, there was no commodity eye-tracking device such as Tobii (Tobii Technology, https://www.tobiipro.com/product-listing/tobii-pro-x2-30/) (accessed on 21 May 2021). Therefore, most gaze studies have focused on improving the accuracy of the eye-tracking technology. Fundamental research on calibration, head-free, and the usage of various cameras have made eye-tracking technology more stable. Since then, many researchers have started to concentrate on analyzing gaze data statistically rather than eye-tracking technology as it is possible to obtain relatively stable gaze data. In particular, trends in the gaze analysis research have been changed as Tobii and SMI (iMotions) (SMI Eye Tracking Glasses—iMotions, https://imotions.com/hardware/smi-eye-tracking-glasses/) (accessed on 21 May 2021) produce eye-trackers, eye-gaze analysis tools, and professional SDKs. Moreover, these tools and SDKs provide basic gaze data visualization such as scanpath, AoI-based, and heatmap.

### 2.1. Gaze Data Visualization

Gaze behavior analysis researchers mainly examine eye movement data using statistical techniques [19,20,21]. Gaze data visualization is utilized independently or in combination with statistical techniques to obtain additional insight. Blascheck et al. [34] summarize common technologies on the gaze data visualization. The most ordinarily used visualization techniques include scanpath, AoI-based, and heatmap. However, the gaze data contains much more information that cannot be analyzed appropriately only with the above visualization techniques. Therefore, researchers have begun to study various approaches. Kurzhals et al. [26] analyze the flow of gaze-concentrated areas by proposing an image-based visualization with gaze stripes. In work by Song et al. [35], GazeDX was shown to enable efficient and comprehensive gaze pattern comparisons. Burch et al. [36] attempted to reduce the complexity of the gaze data analysis by proposing clutter-free color-band visualization with time-varying (x;y) coordinates. Moreover, Blascheck et al. [24] proposed a visualization technique for the gaze comparisons of common and non-common patterns with various AoIs. Fuhl et al. [37] presented an automatic RoI generation algorithm applicable to different eye-tracking datasets to improve manual RoI annotation. Many researchers show how information is perceived through the eyes and how the gaze can be a rationale to evaluate the efficiency of visualization techniques [38,39,40,41]. Ho et al. [42] presented the effectiveness of 2D flow visualization techniques using the eye-tracker to evaluate how every individual perceives the visualizations differently. Existing technologies utilize various visualizations to analyze the gaze.

### 2.2. Visual Saliency

Pioneering research for the visual saliency proposes a model of saliency-based visual attention for the rapid scene analysis [6]. The visual saliency map plays an important role in visualization to emphasize the potential human attention that can be categorized as features. In the gaze analysis study, visual saliency is also applied to automatically generate AoI because AoI annotation is a passive task that needs to regard human visual attention [43]. Itti et al. [6] calculated the saliency map using intensity, color, and orientation. Saliency features are extracted as a feed-forward mechanism from natural outdoor visual stimuli. Jänicke et al. [15] provided a guide for the visual attention to relevant parts that can be applied in visualization areas. They proposed a salience-based quality metric by adjusting color, intensity, and orientation elements. Since the gaze and the visual saliency are closely related to the human perception, Matzen et al. [41] proposed a new visual saliency model, Data Visualization Saliency Model (DVSM), and presented the relationship between the visual acuity and the visual salience. Judd et al. [44] analyzed the gaze data from 15 viewers on 1003 images to answer the questions that most saliency approaches do not match the actual eye motions because the bottom-up computation is employed for the top-down image semantics. In addition, various saliecny features such as entropy [8], curvature [9], and log-spectrum [7] are used for computational saliency models. Geisler et al. [45] create a saliency map by combining Graph-Based Visual Saliency (GBVS) with gaze data. Moreover, there are studies on the gaze in connection with deep-learning [46] and quality evaluation through the gaze tracking data [16].

In this section, we review existing gaze data analysis techniques for gaze data analysis and studies related to visual attention. Table 1 summarizes the eye movements data, gaze visualization, visual saliency, and saliency features used in various studies.

In most studies, visual saliency is applied by showing a saliency map of a stimulus or by combining it with the gaze data. Additionally, although low-level saliency features are essential for saliency model design and eye movement data analysis [32], most studies have focused only on presenting saliency maps. In this paper, we examine human visual attention by proposing a novel gaze data visualization to analyze saliency features and gaze data reciprocally. Our study is the first step towards a visualization study that intuitively presents gaze data and saliency features. We choose low-level saliency features that are important for understanding eye movements to analyze the effectiveness of the proposed visualization [32]. The saliency features include intensity, color, and orientation, and their contribution to the gaze behavior analysis has been validated in feature integration theory [5] and many behavioral studies. Note that our visualization is not limited to intensity, color, and orientation.

## 3. Fixation Identification and Extraction Saliency Feature

**Figure 1:**
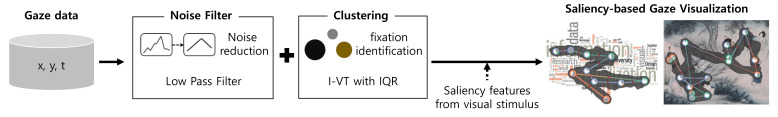
An overview of the data processing. The gaze data and saliency features are visualized together in the saliency-based gaze visualizations. Saliency features are extracted from visual stimulus.

This section describes the eye-tracking data collection environment, fixation identification, and saliency feature extraction techniques. We introduce an overview of our data processing as illustrated in Figure 1.

### 3.1. Environment

We collected gaze data including spatial coordinates and time from the gaze tracking device Tobii pro X2-30 (40 Hz). We developed a framework for the data collection with C++, Qt5 for GUI, and Tobii Pro SDK (Tobii Pro SDK, https://www.tobiipro.com/product-listing/tobii-pro-x2-30/) (accessed on 21 May 2021) to trace the gaze on Windows 10. During the data collection, all observers were given the same environment, such as a 32-inch monitor (3840×2160 resolution), the same position and height of the desk and chair, the same distance between the observer and the monitor, and the same luminance.

### 3.2. Fixation Identification

The collected gaze data mainly contain noise, which is a high-frequency signal [49]. Therefore, it is necessary to remove noise from the gaze data. However, since the noise reduction filter of the gaze data is not strictly defined [50], we use a Low Pass Filter (LPF) that is widely used for the high-frequency signal cancellation. After removing the noise from the data, we identify gaze fixations. We distinguish between fixation and saccade using I-VT (Velocity-Threshold Identification) algorithm [51]. I-VT divides fixation and saccade based on the velocity threshold, which has been applied in various studies due to the simple algorithm using only one parameter. However, in the I-VT algorithm, “blips”, which erroneously classify eye movements into several fixations near the velocity threshold, occur. Therefore, we use IQR (Interquartile Range) [52] to aggregate several fixations that are incorrectly classified as “blips”. We apply the $Dist_{min}$ in the I-VT algorithm to reflect the time information of the gaze data. Note that $Dist_{min}$ is calculated as Q3·1.5·IQR, where IQR=Q3−Q1, and Q1 and Q3 indicate the distances of the first quartile (25%) and the third quartile (75%) gaze data from the center of the data distribution, respectively. If the Euclidean distance of two consecutive points, $P_{i}$ and $P_{\left(i+1\right)}$, is greater than the minimum distance, $Dist_{min}$, it is determined as a saccade.

### 3.3. Extraction Saliency Feature

Besides the gaze fixations, we also extract the saliency features from the visual stimulus. Visual saliency is a study to find a noticeable area in the visual stimulus. Human eye movements and visual attention are affected by visual saliency and cognitive controls such as memory and knowledge. The saliency features are used to generate the saliency map by calculating visual saliency in various computational saliency models. Saliency map proposed by Itti et al. [6] is composed of weighted color, intensity, and orientation features as defined in the following.

$$S=\lambda_{i}F_{i}+\lambda_{c}F_{c}+\lambda_{o}F_{o},$$

where *S* is the saliency map proposed by Itti et al. [6]. $\lambda_{i}$, $\lambda_{c}$, and $\lambda_{o}$ are the weights of intensity, color, and orientation features, respectively. $F_{i}$, $F_{c}$, and $F_{o}$ are the feature maps for intensity, color, and orientation, respectively. $F_{i}$, $F_{c}$, and $F_{o}$ are obtained using dyadic Gaussian pyramids [53]. The nine spatial scales include 1:2${}^{0}∼$1:2${}^{8}$, and each feature map is obtained by the difference between a fine scale ($f_{s}$) and a coarser scale ($c_{s}$). Six intensity maps, $F_{i}$, are computed as $F_{i}\left(f_{s},c_{s}\right)=\Delta I=\left|I\left(f_{s}\right)-I\left(c_{s}\right)\right|$, where $f_{s}\in \left\{2,3,4\right\}$ and $c_{s}=f_{s}+\delta$, δ∈{3,4}. Color maps are calculated as $F_{c}\left(f_{s},c_{s}\right)=\Delta RG+\Delta BY$, where R,G,B, and *Y* are values in RGBYI color channels [54]. There are twelve color maps since the number of $\left(f_{s},c_{s}\right)$ cases is six, and two color sets, ΔRG and ΔBY, are used for the computation. The orientation map is obtained by Gabor pyramids as $F_{o}\left(f_{s},c_{s},\theta \right)=\Delta O=\left|O\left(f_{s},\theta \right)-O\left(c_{s},\theta \right)\right|$, where θ∈{0°,45°,90°,135°}, which are the preferred orientation [55]. Note that each feature is stored in a matrix of which the size is the same as the stimulus image size in our work.

## 4. Gaze Data Visualization

In the previous section, we introduced the visual encoding of the saliency features, which allows us to identify the saliency features of the visual stimuli. In this section, we propose a technique for embedding the saliency features of a visual stimulus into the gaze data visualization to understand visual attention of an observer.

**Figure 2:**
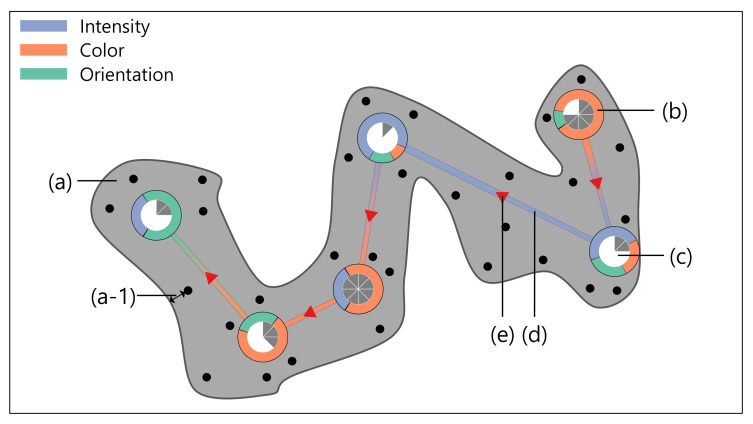
Saliency-based gaze visualization. (a) is the field of view. The raw gaze data points are rendered as the black dots on the field of view. Note that, in the analysis, we show only the field of view. (a-1) is a visual angle degree. The range of visual angle degree is 1 to 2 degrees [56] of the raw gaze point. (b) is the fixation with the saliency features. Intensity, color, and orientation are encoded in blue, orange, and green, respectively. The chart in (c) represents the fixation time with the pie slices. (d) is the link between fixations. The link color indicates the prominent saliency feature. (e) represents the gaze direction.

Figure 2 illustrates the proposed saliency-based gaze visualization, which consists of a field of view in (a), a fixation in (b), a duration time in (c), a link between fixations in (d), and the gaze direction in (e). Note that the black dots rendered on the field of view are the raw gaze data points and that only the field of view is presented in the gaze analysis. The field of view is a foveal area where human visual attention usually focuses, and the visual angle degree is generally 1∼2° [56]. For the implementation of the field of view in (a), we apply the Bubble Sets technique [57]. Since the conventional Bubble Sets are grouped based on the distance between points, the raw gaze points are bundled based on the gaze locations without considering the time information when applied to the gaze data. Therefore, we modify the grouping condition of the existing Bubble Sets algorithm by adjusting the raw gaze points order and distance threshold so that only the close raw gaze points are grouped spatiotemporally. We draw the area 1∼2° away from the raw gaze point as the field of view in (a-1). In (b), we show the prominent saliency feature and the percentage of saliency features at the fixation. The color encoding includes intensity, color, and orientation as blue, orange, and green, respectively. We crop the area image corresponding to the field of view of gaze points aggregated around fixation and extract the saliency features from the cropped image (See Section 3). The extracted saliency features are stored in a 2Dd matrix of the same size as the cropped image. The average of each saliency feature matrix is used as the saliency feature value of fixation. (c) presents a pie chart indicating the fixation duration. We normalize the durations in the fixations and present the relative durations for the comparison. In (d), the line width is determined by the value of the prominent saliency feature. Line color is represented by interpolating the colors encoded in the prominent saliency features of source and destination fixations. In (e), the arrow color is chosen as red to highlight the gaze direction. Thus, in the visualization, we provide analysts with more intuitive visual clues for both the saliency features and the saliency feature ratio.

## 5. Gaze Analysis

In this section, we apply the saliency features as a visual clue to competitive visual attention selection of an observer to analyze gaze data. We analyze the gaze behavior using the saliency-based gaze visualization. For the data collection, we recruited 23 participants. There were seventeen males and six females, with an average age of 28 (standard deviation, SD = 3.67). The average knowledge level about visualization was 2.17 (SD = 1.53), where 0 is none and 4 is the professional level. The average knowledge level about the eye-tracking technology was 1.57 (SD = 1.24). We gave the participants a description of the experiment before data collection and interviewed them about their medical characteristics, especially those related to the eyes. Through the interview, it was found that one participant had *red-color weakness*. After the data collection, all participants were given a 10-second free-viewing task. At the end of data collection for each case study, we recorded the eye movements, intentions, and visual stimulus that the participants remembered. In addition, a 5-minute break was equally provided between data collections. We informed all participants that they could rest for more than 5 minutes if they felt tired, but no one took additional rest. After all data collection was completed, a questionnaire survey was given to compare the existing gaze data visualizations with the proposed visualizations. We also received feedback from the participants. Table 2 summarizes the number of records of eye-tracking data collected from the participants.

### 5.1. Gaze Analysis with Saliency Features

**Figure 3:**
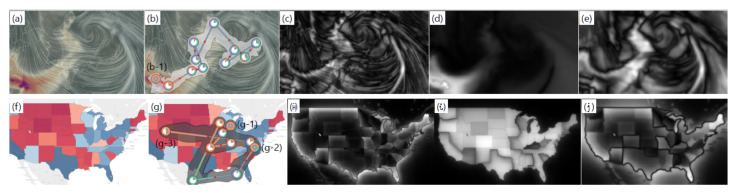
Gaze behavior analysis with the saliency features. (**a**,**f**) are the visual stimuli. (**b**,**g**) are the saliency-based gaze visualizations. (**c**,**h**) are the intensity feature map. (**d**,**i**) are the color feature map. (**e**,**j**) are the orientation feature map.

We analyze the gaze behaviors with the saliency features in Figure 3. (a) is the visual stimulus that is a visualization of fine dust density and wind flow as a heatmap and a line integral convolution, respectively (Earth Nullschool, https://earth.nullschool.net/) (accessed on 21 May 2021), whereas the visual stimulus in (f) is an example of a geo-visualization created in Tableau (Tableau Sftware, https://www.tableau.com/) (accessed on 21 May 2021). Figure 3 (c) and (h), (d) and (i), and (e) and (j) are the intensity, color, and orientation feature maps for two visual stimuli, respectively. (b) and (g) show the saliency-based gaze visualizations of a participant for the visual stimuli, respectively.

In the visual stimulus analysis presented in Figure 3d, the color saliency feature is concentrated in the lower left area. As seen in (b), the participant moved his gaze from the high dust density area to the low dust density area. After identifying areas of high fine dust density, this participant followed the fine dust spreading from left to right. In this analysis, we can observe that the fixation time in the region (b-1) where the color feature is prominent is long whereas the fixation time in the region containing less color features is short. We want to understand whether this gaze behavior is due to the participant being sensitive to the color saliency feature or whether they have a habit of viewing the first area they see within the visual stimulus. However, the visual stimulus in (a) lacks clues for the analysis since the color feature is found only in specific areas, which could cause a biased analysis. Thus, we showed a new visual stimulus to the participant, as presented in Figure 3f. The visual stimulus has three saliency features more uniformly compared to (a), and there are also particularly noticeable areas only with the color feature as shown in (i). The gaze behaviors of the same participant are visualized in both (g) and (b). The fixations are mostly located in the prominent area only with the color feature, and the fixation time is long in the areas (g-1), (g-2), and (g-3). Additionally, the fixation time is short in the remaining areas. Therefore, in this analysis, we can infer that the participant responds sensitively to the color feature among the saliency features. The extension of this case study can be utilized to produce effective visualization by analyzing the effect of saliency features in data visualization on gaze behavior in the future.

### 5.2. Visual Search Analysis with Visual Attention

**Figure 4:**
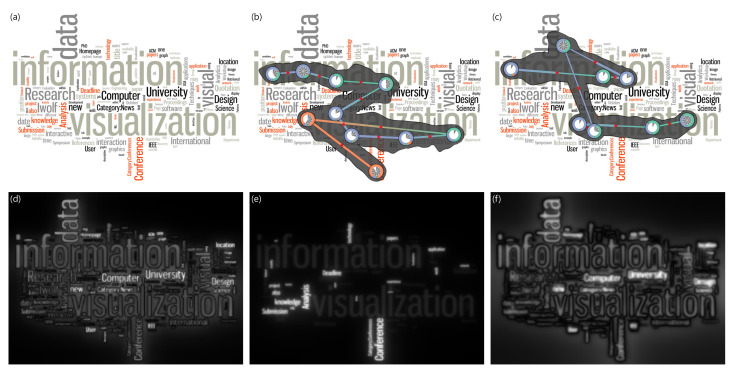
Gaze analysis on the tag cloud visualization. (**a**) is the visual stimulus. (**b**,**c**) are the saliency-based gaze visualizations of two participants. (**d**–**f**) are the intensity, color, and orientation feature map, respectively.

In this case study, we analyze the influence of visual attention on gaze movements such as the visual search task. In this experiment, the tag cloud visualization is set as a visual stimulus. Figure 4a is the visual stimulus, whereas, (d), (e), and (f) are the intensity, color, and orientation feature maps, respectively. In (b) and (c), we compare two gaze datasets with the same visual stimulus but with different responses from two participants.

We hypothesize that *the visual search task is different depending on the saliency features that an observer responds to*. We collected the gaze data from two participants. We also asked the participants to mark the most prominent words from the visual stimulus for 10 seconds. In this experiment, we compare the data of two participants with and without the *red-color weakness* to control only the color features that the observers respond to in the same environment. (c) presents the gaze visualization of a participant having a *red-color weakness*. In the post-experiment interview, the participant in (b) mentioned that *information, visualization*, and *conference* are the most memorable words, while the participant in (c) specified *data, information*, and *visualization*. The visual stimulus in Figure 4a is a tag cloud visualization including keywords related to the information visualization. The intensity feature in (d) and orientation feature in (f) are prominent in this visual stimulus, whereas the color feature in (e) is relatively less prominent.

It is seen that both participants in Figure 4b,c have the same habit of moving their gaze from left to right and from top to bottom when searching for information. However, the fixation interval of the participant in (b) is consistent except for the last two. On the other hand, two major fixation patterns in (c) are formed together repeatedly as if the gaze jumps at a distance. Therefore, the gaze behaviors of both participants have similar patterns in the directions of the overall movements, but the gaze movements such as the speed of reading the text and the amount of gaze fixation time in an area are different.

Both participants commonly remembered *information* and *visualization* as the prominent words, but the participant in (b) referred to *conference* and the participant in (c) referred to *data* as the most memorable word, respectively. To find the reason for the difference, we can utilize our gaze visualization with the saliency features. The saliency feature compositions of the two words are different. The most striking saliency feature in the area containing *data* is the intensity feature in (d) and orientation feature in (f), while the color feature in (c) is most prominent in the area containing *conference*. The participant in (c) responded sensitively to the intensity and orientation features rather than the color feature. Thus, they seemed to remember the word *data* since it contains a large amount of intensity and orientation features. On the other hand, the participant in (b) responded to all three features. They could not recognize the word *data* since *data* is not obviously prominent in terms of the saliency features while they explored the visualization. The participant in (b) continued to discover the information even after seeing the word *visualization*. Therefore, they found and remembered the word *conference*. When comparing the differences between these two participants, the participant with the *red-color weakness* in (c) did not respond to the color feature as compared to one without the *color weakness* in (b). The extension of this case study can be utilized for effective visualization design using saliency features. In particular, when given the same cognitive control as a task or memory, it is possible to analyze the effect of saliency features on a searching task.

### 5.3. Influence of the Saliency Features on Gaze Movement

**Figure 5:**
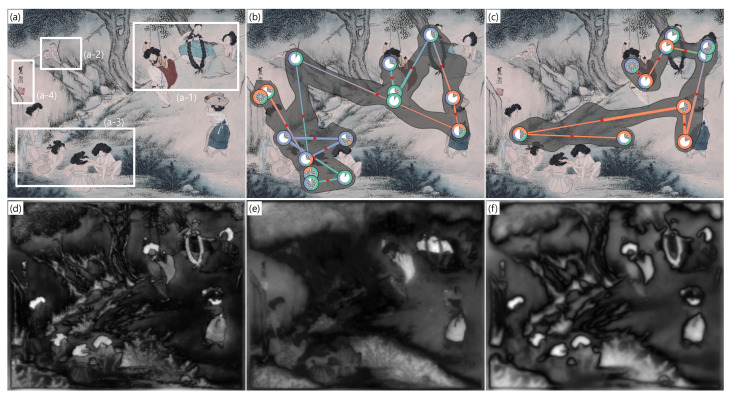
Gaze analysis on a painting. (**a**) is the visual stimulus. (**b**,**c**) are the saliency-based gaze visualizations of two participants. (**d**–**f**) are the intensity, color, and orientation feature map, respectively. The visual stimulus in (**a**), “A Dano festival”, is provided by Kansong Art and Culture Foundation in Seoul, South Korea.

In this case study, we analyze the influence of the saliency features on the gaze movement. Figure 5 shows an example of the painting as a visual stimulus. (a) is the visual stimulus. (d), (e), and (f) shows the intensity, color, and orientation feature maps on the visual stimulus, respectively. We compare the gaze data of two participants who viewed the visual stimulus with different searching behavior in (b) and (c), respectively. Figure 5a is a Korean masterpiece from the 18th century, and the painter emphasized colors such as red, yellow, and blue, in the painting. Nevertheless, the children were drawn relatively invisible. As shown in (a-2), the children are not well distinguished from the surrounding rocks. The reason is that the intensity feature in (d) and orientation feature in (f) compose the saliency for both the children and the surrounding rocks. Since both saliency features for the children and the rocks are same, it is hard to discover the children unless we focus on them. On the other hand, the women are relatively easy to spot since they are depicted relatively colorful with the color feature in (e), unlike the surrounding background.

We hypothesize that *the results of a visual search task depend on saliency features that an observer responds sensitively*. (b) and (c) present the gaze data visualizations of two participants telling the most contradictory conclusions in the interviews after the data collection. In the interview, we have asked the participants to describe the painting. The participant in (b) described in detail as *“I saw a woman playing on a swing in (a-1). There were a few more women around. I found children hiding on one side in (a-2). After a while, I noticed that the children saw women wash their hairs in (a-3). Finally, I saw that there are a signature and an official stamp of the painter on the left side of the painting in (a-4).”* On the other hand, the participant in (c) said, *“There were a few women around the girl on the swing in (a-1). There were also women washing their hairs in the valley in (a-3)”*, which is relatively brief.

In this case study, we also compare the gaze data of two participants. Both participants have fixations on the people as seen in Figure 5b,c. Therefore, we can see that they both have a common habit of searching for people-oriented objects. The difference is that the participant in (b) has more fixations formed than the participant in (c), such as the hidden children in (a-2), the fixations on the women in (a-3), and the signature and official stamp of the painter in (a-4). Therefore, both participants tend to search for people-oriented objects in the paint but have different observation habits. Comparing the visualizations of Figure 5b,c, we find that the participants focus on different saliency features. The participant in (b) focuses on the three saliency features evenly. Thus, in the gaze visualization, all three saliency features are shown uniformly at the fixations and many fixations are formed. Additionally, the gaze fixations of the participant in (b) are distributed over a wider range of the visual stimulus than ones of the participant in (c) and there are more fixations in (b) than ones in (c). This indicates that there is much information to be processed and they explored and received the information minutely. On the other hand, fewer fixations are formed in (c), and they are mainly composed of the color feature. Therefore, we analyze that the participant in (b) viewed more scope and more detail than the participant in (c). Additionally, in the interviews of the two participants, there was a qualitative and quantitative difference in describing the painting. It is also possible to understand why the participant in (b) suddenly came to see the picture more closely by deducing the purpose of gaze. After the participant saw the area where the children are hiding, the number of fixations has increased. Additionally, they seemed to scan more objects to discover the reason why the children are hiding. Therefore, it can be understood that the description of the participant in (b) was detailed in the interview. In this analysis, we can observe that the participant in (b) who utilizes various saliency features for the information selection showed more detail visual search tasks than the participant in (c) who focused only on the color feature. The extension of this case study can be utilized in a study to analyze the effect of the saliency feature on the searching task under the same cognitive control environment.

**Figure 6:**
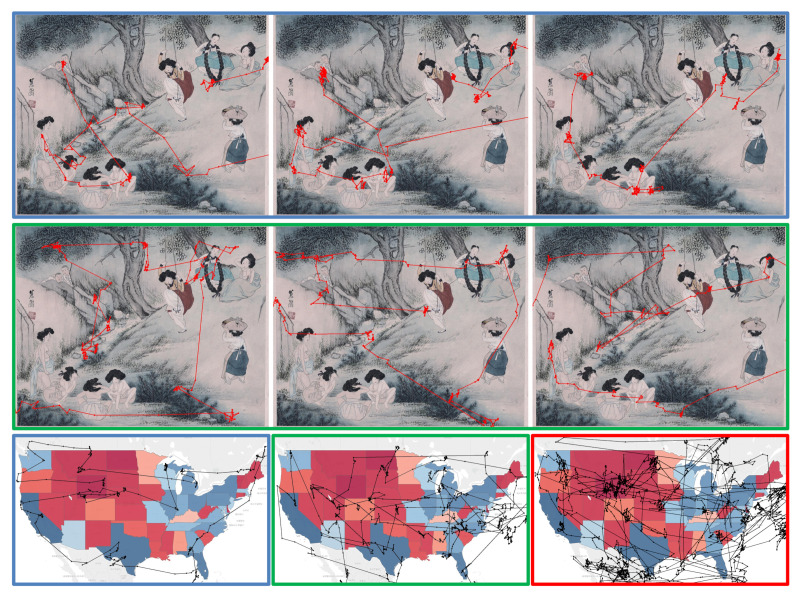
Comparison of gaze data for three participants. The scanpath of each participant is identified by blue, green, and red box frame colors.

**Figure 7:**
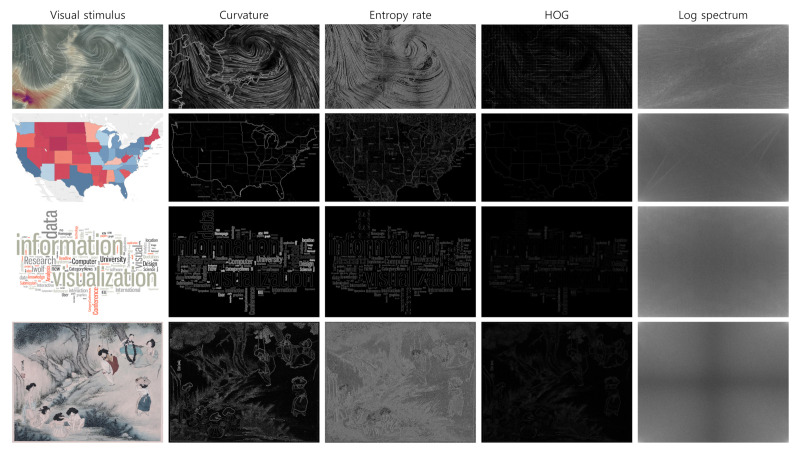
Feature maps extracted from various visual stimuli. Features maps include curvature [9], entropy rate [8], histogram of oriented gradients (HOG) [11], and log spectum [7].

We compared the gaze data to avoid participant characteristics from affecting the analysis. Figure 6 represents the gaze data of three participants with scanpath visualizations. The blue, green, and red box frame colors depict three participants. We used the data from the blue and green participants in Figure 6, showing different eye movement patterns depending on the visual stimulus to avoid participant characteristics from affecting the analysis. The data, such as the red frame in Figure 6, which always shows the same scanpath regardless of the visual stimulus, were not used. However, it is difficult to say that this data selection excluded the characteristics of all participants. Therefore, we plan to consider filtering or models to exclude participant characteristics in future work. Figure 7 shows feature maps other than intensity, color, and orientation extracted from visual stimuli used in all case studies. The saliency feature maps shown in Figure 7 include curvature [9], entropy rate [8], histogram of oriented gradients (HOG) [11], and log spectum [7].

### 5.4. User Study

In this section, we evaluate our gaze visualization techniques compared with the existing techniques through the survey after the experiments. We compare the point-based, heatmap, AoI-based, scanpath, and our saliency-based gaze data visualization. The participants were given the same analysis assignments, and each visualization was scored with the 10-point Likert Scale (0: I do not understand, 10: I understand) for the following eight questions. The Table 3 summarizes the mean and standard deviation of the responses to each question per visualization from 23 participants.

Q1: Can you use the gaze distribution in the gaze analysis?
Q2: Can you follow the gaze flow in the time order?
Q3: Can you find the area in which the participant is interested?
Q4: Can you spot an area where the gaze stayed for a long time?
Q5: Can you reason the gaze concentration by viewing only the visual stimulus?
Q6: Can you analyze how the participant viewed to understand the visual stimulus?
Q7: Can you discover observer characteristics in the given visualization?
Q8: Can you analyze how the eye movement is related with the visual stimulus in the given visualization?

After the above survey, we selected five participants who had expertise and asked for in-depth interviews to gather feedback. p1 is a participant who majors in data visualization and has experience in eye tracking. p2 to p4 are participants who major in data visualization. p5 is a doctoral graduate who majors in computer vision with knowledge of saliency features. The comments received from the participants are summarized as follows.

- Point-based: We can see the gaze distribution easily, but it is difficult to analyze the meaning of the gaze by only the point visualization (p1, p2). This visualization also interferes with the analysis since it blocks the visual stimulus (p1, p2). However, it is easy to understand the visualization due to the simplicity (p5).
- Heatmap: This is the visualization that we often see (p5). It seems to be helpful to discover what the gaze focuses on (p1, p2, p3, p4, p5). However, the scope of analysis seems to be limited (p1, p3).
- Scanpath: This is also a visualization that we often see (p5). The gaze movement can be analyzed easily according to the time flow, and the shift of interest can be extracted (p1, p2, p5). However, the analysis is limited, and when the gaze becomes complicated, it seems difficult to analyze (p1, p3, p4, p5).
- AoI-based: It seems to be a useful visualization for unfamiliar information analysis (p1, p2, p3). However, it does not seem to be very helpful for the information already known (p1, p2). It would be helpful if we could see the gaze flow together with this visualization (p1, p3).
- Saliency-based: This visualization seems confusing at first because it shows other information than the existing visualizations, but after learning the meaning of the information provided, it was easy to analyze the visualization (p1, p4, p5). It was possible to know the fixation range using the field of view, but it was difficult to actively utilize it in the analysis (p5). This visualization is efficient because it can intuitively identify saliency features without additional data (p1, p5). In addition, by using saliency features, it is possible to analyze gaze behavior from more diverse viewpoints (p1, p5).

## 6. Limitations and Discussion

In this section, we present the concerns that may arise when using the proposed gaze visualization. These need to be considered in order to minimize faulty analysis results.

### 6.1. Fixation Clustering

In Section 3, we applied I-VT with IQR as an identification algorithm for the fixation. However, there are many techniques to identify fixations, such as I-DT, I-HMM, I-MST, I-AOI [51], K-means [58], Mean-Shift [59,60], DBSCAN [61,62], Graph-based joint [63], Distance-Threshold [64], and Projection clustering [65]. Each fixation identification algorithm makes a group of raw gaze points differently as a fixation. Therefore, different analysis results may be produced due to the fixation identification algorithms. In this paper, we did not address problems that may arise due to differences in fixation identification algorithms. We plan to compare fixation identification algorithms and to find the most suitable algorithm for visual attention analysis in the future.

### 6.2. Field of View

In the proposed saliency-based gaze visualization, the field of view of an observer is presented in a transparent gray area, which provides intuitive visual clues to the analyst. The field of view appears to be visualized automatically from raw gaze data. However, the field of view is an open observation area viewed by a human and has different ranges depending on persons [56,66]. It is also difficult to determine which point in the raw gaze data is noise. Therefore, postprocessing tasks are needed, such as comparing the raw gaze data with interviews after the data collection and setting the range of the field of view. However, since human judgment is not always perfect, the possibility of error should be considered in the analysis.

### 6.3. Fixation Overlaps

We visualized the gaze data and saliency features together to provide information to analysts. However, collecting the gaze data over a long time causes the overlaps of fixations and links between fixations due to the increased number of fixations or complicated gaze movements. Since the data overlap interferes with the gaze analysis, we permitted the participants to explore the stimuli within a minimum amount of time in the experiment in order to avoid the overlap effects. Therefore, in all experiments, the data collection time was limited to less than 10 seconds to avoid overlapping to the extent that data analysis was not possible. However, this time restriction is one of the controls for the experimental design. Controlling the experimental design can interfere with various approaches to the gaze data analysis. Therefore, the limitation caused by the overlap in the gaze data analysis needs to be continually addressed in the future. Additionally, we should keep it in our mind that, if the time constraints disappear in the experimental design in the case studies, the analysis results might be different from the present ones.

### 6.4. Saliency Features

In our proposed visualization technique, the saliency features of visual stimuli are very important visual clues for the gaze data analysis. However, when using the saliency features in the analysis, faulty analysis results can occur when a certain saliency feature become prominent across the entire area of a visual stimulus. For example, if an intensity feature appears entirely in a visual stimulus, the analyst would always be provided with a visual stimulus for the intensity prominence at all fixations. This problem makes it difficult to determine whether the gaze movement is affected by the intensity feature. Thus, although the saliency features are good visual clues for analyzing the gaze behaviors, we should admit to the possibility of errors. We examined whether intuitive visualizations of eye movement data and saliency features help analyze gaze behavior. We utilized only intensity, color, and orientation features in our analysis, on which human visual perception is strongly affected. However, many additional saliency features can be used, as reviewed in Section 2. However, as the number of saliency features displayed in visualization increases, intuitive visual encoding becomes challenging. Therefore, it is necessary to investigate a model that recommends saliency features for analysis according to visual stimulus. We also used blue, orange, and green colors to represent intensity, color, and orientation, respectively. This color combination is not an optimal combination for detecting color overlaps or patterns. However, for the optimal color selection, there are many factors to consider, such as color combinations, analyst preferences, and the impact on cognition. Note that we represented the gaze direction as a red arrow in the gaze data visualization. Red is not the optimal color but is used as a prominent color to emphasize the meaning. Therefore, we plan a design study for the optimal color choice.

## 7. Conclusions and Future Works

In this paper, we proposed a novel visualization technique embedding the saliency features for analyzing the visual attention of an observer through the gaze data. Our proposed visualization provides intuitive visual clues for the saliency features such as intensity, color, and orientation of visual stimuli in the gaze data analysis. We analyzed the gaze behavior using the saliency-based gaze visualization in the case studies and evaluated how our approach to embedding saliency features within the gaze analysis supported us to understand the visual attention of an observer.

In this study, we controlled the noise caused by the human and environmental factors and the gaze data clustering so that they did not affect the analysis results. Therefore, the parameters of the noise filter and the clustering algorithm were adjusted depending on the interviews received from the participants during the gaze data collection. However, it is not always possible to rely on the interviews in the gaze analysis. Additionally, determining the parameters of the noise filter and the clustering algorithm is an essential issue in the gaze data analysis. We, therefore, plan to address this issue in the future study. In our study, we only examined the gaze data collected from static visual stimuli. Since visual stimuli can become dynamic [34], we will study a methodology for analyzing dynamic visual stimuli similar to the research by Kurzhals et al. [26]. Lastly, in addition to the intensity, color, and orientation used in the model Itti et al. [6], we plan to analyze various saliency features, such as log spectrum [7], entropy [8], curvature [9,10], histogram of oriented gradients [11,12,13], and center-bias [14]. Moreover, we plan to examine a model that recommends suitable saliency features for analysis depending on visual stimuli and more effective visual encoding techniques.

## Figures and Tables

**Table 1:** Summary of related studies. The table shows the eye movements data visualization and measures, visual saliency visualization and features, and visualization style used in each study.

	Study	Eye Movements		Visual Saliency		Style
		Visualization	Measures	Visualization	Saliency Feature	
Gaze visualization	[26]	point-based, scanpath, AoI-based, space-time cube	x, y, time, stimulus	-	-	-
	[35]	point-based	x, y, stimulus	-	-	-
	[36]	new style (AoI-based)	x, y, duration	-	-	-
	[24]	new style (AoI-based)	transitions, duration	-	-	-
	[22]	heatmap	x, y, duration, RoI, stimulus	-	-	-
	[19]	-	fixation number, duration	-	-	-
	[20]	-	EMP (Eye movement perimetry), SRT (saccadic reaction time)	-	-	-
	[21]	scanpath, AoI-based	x, y, AoI, duration	-	-	-
Visual saliency	[6]	scanpath	x, y, duration, stimulus	saliency map	intensity, color, orientation	separate
	[15]	-	-	saliency map, contribution map	intensity, color, orientation	-
	[41]	-	x, y, duration, stimulus	saliency map	color, text-specific	-
	[44]	point-based, heatmap	x, y, RoI	saliency map	intensity, color, orientation, center, horizontal line, face, person	overlap
	[46]	point-based	x, y	saliency map	data-driven	separate
	[8]	heatmap	x, y	saliency map	entropy	separate
	[9]	-	reaction time	-	curvature	-
	[7]	-	-	saliency map, object map	log spectrum	-
Both	[45]	scanpath, heatmap	x, y, duration, stimulus	heatmap	texture (GBVS [47,48])	separate
	our proposal	new style (scanpath)	x, y, duration, stimulus	-	intensity, color, orientation (not fixed)	combined

**Table 2:** Number of eye tracking data records collected from 23 participants.

	Case Study 1		Case Study 2	Case Study 3
	Visual Stimlus 1	Visual Stimulus 2	Visual Stimulus 3	Visual Stimulu 4
All records number	8855	12,995	7981	6256
Average records number	385	565	347	272

**Table 3:** The average scores for point-based, heatmap, AoI-based, scanpath, and saliency-based gaze data visualization.

		Point-Based	Heatmap	AoI-Based	Scanpath	Saliency-Based
Q1	mean	7.74	7.67	3.09	5.87	7.67
	SD	2.24	2.70	3.36	2.93	2.80
Q2	mean	3.39	2.96	1.44	4.92	8.35
	SD	3.45	3.36	2.31	3.30	2.89
Q3	mean	7.40	7.13	2.57	4.26	7.78
	SD	2.87	2.65	3.40	3.57	3.22
Q4	mean	6.44	6.57	7.14	2.78	7.70
	SD	2.84	3.09	2.65	3.23	3.10
Q5	mean	6.83	6.29	2.48	4.46	7.13
	SD	2.96	2.73	3.25	3.62	3.31
Q6	mean	6.13	5.87	3.83	5.00	6.91
	SD	3.05	2.67	3.66	2.83	3.19
Q7	mean	3.52	3.30	1.48	4.00	5.52
	SD	3.10	2.91	2.40	3.26	3.67
Q8	mean	5.91	5.30	2.17	2.22	6.48
	SD	3.01	3.48	3.05	2.86	3.65

## Data Availability

Not applicable.
